# Predictors of Neonatal Mortality in Ethiopia: A Comprehensive Review of Follow-Up Studies

**DOI:** 10.1155/2022/1491912

**Published:** 2022-02-11

**Authors:** Derara Girma, Hiwot Dejene, Leta Adugna

**Affiliations:** Public Health Department, College of Health Sciences, Salale University, Fitche, Ethiopia

## Abstract

**Background:**

Neonatal mortality remains a prominent public health problem in developing countries. Particularly, Ethiopia has a higher neonatal mortality rate than the average sub-Saharan African countries. Hereafter, this review article was aimed at synthesizing existing predictors of neonatal mortality in Ethiopia.

**Methods:**

A systematic search and review of peer-reviewed articles were conducted on the predictors of neonatal mortality in Ethiopia. A search of key terms across different databases including Web of Science, SCOPUS, Cochrane Library, PubMed, EMBASE, Hinari, and Google Scholar was conducted, supplemented by reference screening. The SANRA tool was used to critically appraise studies included in the review.

**Results:**

After removing duplicates and applying the eligibility criteria, 14 of the 64 initially identified articles were included in the final review. These were original articles published between 2011 and 2021. The identified predictors were narrated and presented under different domains. Accordingly, sociodemographic predictors such as residence, distance from the health facility, and maternal age; service delivery-related predictors such as no ANC follow-up, not taking iron-folic acid supplementation during pregnancy, and no PNC visit; neonate-related predictors such as low birth weight, extreme prematurity/preterm, and low APGAR score; pregnancy and childbirth-related predictors such as birth interval < 18 months, twin pregnancy, and time of rupture of membrane > 12 hours; and maternal-related predictors such as maternal HIV infection, maternal childbirth-related complications, and maternal near-miss were stated to increase a likelihood of newborn death in Ethiopia.

**Conclusion:**

Public health interventions directed at decreasing neonatal mortality should address the rural residents, mothers not having ANC follow-up, low birth weight, twin pregnancy, and maternal HIV infection. The wealth of data gathered during primary research should not only lead to identification of predictors, but should also provide guidance for health system intervention strategies in a country aiming to reduce neonatal mortality.

## 1. Introduction

Neonatal mortality rate is the number of deaths during the first 28 completed days of life per 1,000 live births (LBs), and it remains a serious global public health problem [1]. Globally, 2.4 million children died in the first month of life in 2019. Approximately, there are 6,700 newborn deaths daily, contributing to 47% of all child deaths under the age of 5 years, up from 40% in 1990. Specifically, sub-Saharan Africa (SSA) had the highest neonatal mortality rate in 2019 at 27 deaths per 1,000 LBs, followed by Central and Southern Asia with 24 deaths per 1,000 LBs [2]. To tackle these figures, Sustainable Development Goal (SDG) targets a reduction in neonatal mortality to at least 12 per 1,000 LBs by 2030 [3].

Ethiopia has attained prominent attainments in improving the health status of children in the last two decades. Between 1990 and 2015, child deaths have diminished by two-thirds. The under-5 mortality rate decreased from 123 per 1,000 LBs in 2005 to 59 in 2019. Similarly, the infant mortality rate decreased from 77 per 1,000 LBs to 47 in 2019. However, neonatal mortality remains high with a modest decline—from 39 deaths per 1,000 LBs in 2000 to 33 in 2019 [4]. Startlingly, according to EDHS reports, there is an increment of neonatal mortality from 29 deaths per 1,000 LBs in 2016 to 33 in 2019 [5, 6]. As a result, neonatal conditions are nowadays among contributors for 58% of Ethiopia's disability-adjusted life years (DALYs) [4].

Currently, Ethiopia has planned to diminish neonatal mortality from 33 per 1,000 LBs to 21 per 1,000 LBs by the year 2024/25 [4]. As a result, understanding the identified predictors linked to neonatal mortality obtained from different primary findings conducted using robust epidemiologic study design, i.e., cohort study, is critical in driving the development of focused and evidence-based interventions to prevent newborn deaths.

Overall, in an attempt to lessen newborn mortality, it is indispensable to recognize and effectively engage on the predictors that have been tremendously established so far [7]. Hence, this comprehensive review of articles was aimed at synthesizing countrywide existing predictors of neonatal mortality in Ethiopia.

## 2. Main Text

### 2.1. Search Strategy

This comprehensive narrative review was conducted by considering SANRA (the Scale for the Assessment of Narrative Review Articles) guidelines [8]. The eligible articles for this review were selected in terms of full-text articles, based on inclusion criteria. The Web of Science, SCOPUS, Cochrane Library, PubMed, EMBASE, Hinari, and Google Scholar were systematically searched for articles. These comprised all fields and Medical Subject Headings (MeSH terms). The studies were accessed using the following search terms: “neonatal mortality,” predictors,” “risk factors,” “neonatal death,” “newborn,” “cohort,” “follow up,” and “Ethiopia.” The search terms were used individually and in combination using “AND” and “OR” Boolean operators. The search was guided by PECO: population neonates (age < 28 days), the occurrence of death before 28 days after birth. The selected articles were then entered into the Mendeley software, and the duplicates were eliminated.

### 2.2. Eligibility Criteria

The following are the eligibility criteria in the study:

*Study design:* articles with cohort study design were included.

*Population:* studies conducted only in Ethiopia were included.

*Language:* all articles published in the English language were included.

*Publication year:* articles published from 2011 to 2021 were included.

### 2.3. Search Results

**Figure 1:**
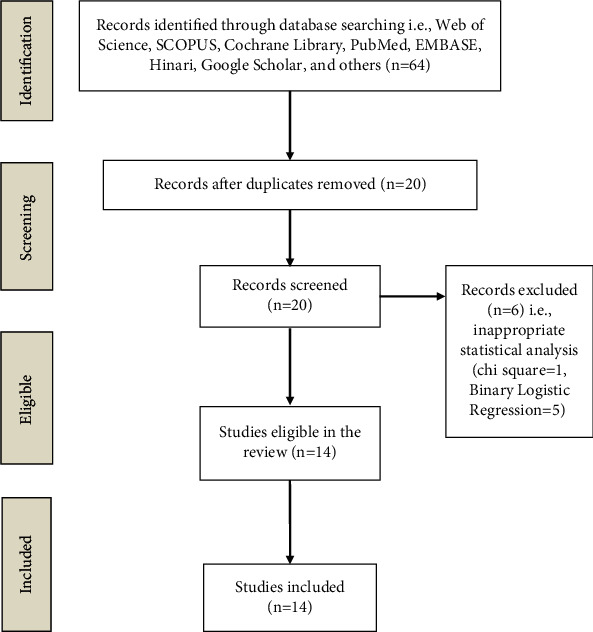
Flow diagram of the review process.

The initial search found 64 studies for this review, but after removing duplicates and applying the criteria listed above, the number of studies was narrowed to 14 articles, all of which are cohort in their study design (Figure 1).

## 3. Predictors of Neonatal Mortality in Ethiopia

Several predictors were identified to increase neonatal mortality in Ethiopia. The most significant predictors are thematized under different domains as the following.

### 3.1. Sociodemographic Predictors

In Ethiopia, different sociodemographic predictors were identified to heighten neonatal mortality. Accordingly, neonatal mortality was higher among newborns whose maternal age is more than 30/≥35 years at birth [9, 10] and among rural resident mothers [9, 11, 12]. Additionally, factors such as the family size of more than seven [9], mothers unable to read and write [10], and mothers being unemployed [13] were identified as major predictors for neonatal mortality. Besides, distance from health facilities [14, 15], low wealth index [15], and low monthly income [16] increases the likelihood of neonatal mortality in Ethiopia.

### 3.2. Service Delivery-Related Predictors

Service delivery is the process of providing a service related to maternal and child health (MCH) to mothers during pregnancy, birth-giving, and after giving birth. Consequently, not having antenatal care (ANC) follow-up [9, 12, 13, 17–20] was identified as the main predictor for neonatal mortality by numerous studies. In addition, not taking iron-folic acid supplementation during pregnancy [9] was found to be among the predictor for neonatal mortality in Ethiopia. Concerning the service that would be provided during newborn delivery, factors such as delivery assisted by a traditional birth attendant (TBA) [9] and neonates born by cesarean section [18] were indicated as chief contributors to neonatal mortality in a country. On the other hand, there is an article that reported cesarean section delivery to be a protective factor for neonatal mortality [19].

Regarding to factors that can increase the probability of newborn death after birth, factors such as neonate resuscitation [18, 20], utilizing of oxygen therapy [20], and mothers who had no postnatal care visit [21] were found as independent predictors of neonatal mortality in Ethiopia.

### 3.3. Neonate/Newborn-Related Predictors

Several neonate/newborn-related predictors influence the probability of their survival in Ethiopia. Thus, a lot of studies were found that newborn low birth weight (<2500 grams) [9, 12, 14, 19, 20] was a significant contributor to neonatal death in Ethiopia. Moreover, inborn neonatal disorders such as extreme prematurity or preterm [11, 12, 20, 22], small size at birth [17, 21], and large size at birth (macrosomia) [17] were the significant predictors for neonatal mortality across a country.

Furthermore, other afterborn neonatal ailments increasing the likelihood of neonatal mortality in Ethiopia were reported to be neonatal sepsis [11], respiratory distress [11] or neonatal admission due to respiratory distress syndrome [13, 20], low APGAR score [11, 16, 19, 20], first minute Apgar score classification of severe [13], hyaline membrane disease [18], perinatal asphyxia [18], hypothermia [19, 20], asphyxia [20], and neonates with other complications [14, 21].

In addition to the neonatal-related predictors stated above, studies also found that neonates who had initiated exclusive breastfeeding (EBF) after an hour [18, 19, 21] and neonates not initiating exclusive breastfeeding at all [13, 14] were more likely to increase the chance of newborn death in a country. As well, different studies noted that being a male newborn [15, 17, 21] is a predictor for mortality in Ethiopia.

### 3.4. Pregnancy and Childbirth-Related Predictors

Our study found that a presence of abortion history and previous sibling death [22] had an increased likelihood of neonatal mortality [9] in Ethiopia. Furthermore, among the pregnancy-related predictors, the occurrence of illness during pregnancy [9], preceding birth interval less than 18 months [17], and twin pregnancy [10, 17, 18, 22] was determined as major predictors for neonatal mortality in a country. In a further complication, childbirth-related predictors such as mothers' time of rupture of membrane (ROM) > 12 hours before delivery [19] and fetal malpresentation [16] were shown to have a positive association with neonatal mortality.

### 3.5. Maternal-Related Predictors

In Ethiopia, a number of articles highlighted significant findings of maternal risk factors for neonatal mortality. Accordingly, findings specified that a history of different medical conditions including maternal diabetic mellitus [11] and positive maternal HIV status [10] was associated with the occurrence of newborn death in a country. In added evidence, various findings described that maternal delivery (childbirth-related) occurrence of complications [14, 20] was revealed as predictors for neonatal mortality. In other aspects, being a primigravida (no previous birth) [22] and a maternal near-miss [16] were stated to increase the likelihood of newborn death in Ethiopia.

## 4. Conclusion

Targeting the identified predictors will assist in achieving the national objective and target set by Ethiopia to reduce neonatal mortality to 21 per 1,000 live births [4]. Public health interventions directed at decreasing neonatal mortality should address the rural residents, mothers not having ANC follow-up, low birth weight, twin pregnancy, and maternal HIV infection. Furthermore, in Ethiopia, where plenty of predictors for neonatal mortality have been identified using a robust epidemiologic design (i.e., cohort design), the future investigation should focus on either implementing an intervention strategy based on previously identified predictors or attempting in adding new predictor(s). Also, the researchers should focus on prospective longitudinal studies to identify the strongest epidemiological predictors of neonatal mortality in the future.

## Data Availability

The data used to support the findings of this study are included within the article.
